# Protocatechuic acid and syringin improve cardiac damage and autonomic imbalance caused by dyslipidemia in mice

**DOI:** 10.3389/fphar.2026.1809711

**Published:** 2026-05-18

**Authors:** Myeongguk Jeong, Yeeun Kim, Hyeokjin Kwon, Kyung-Yae Hyun, Do-Young Kang, Aelee Jang, Yeongdon Ju, Go-Eun Choi

**Affiliations:** 1 Department of Biomedical Laboratory Science, College of Health Sciences, Catholic University of Pusan, Busan, Republic of Korea; 2 Next-Generation Industrial Field-Based Specialist Program for Molecular Diagnostics, Brain Busan 21 Plus Project, Graduate School, Catholic University of Pusan, Busan, Republic of Korea; 3 Department of Translational Biomedical Sciences, Graduate School of Dong-A University, Busan, Republic of Korea; 4 Department of Clinical Laboratory Science, Dong-Eui University, Busan, Republic of Korea; 5 Department of Nuclear Medicine, Dong-A University College of Medicine, Busan, Republic of Korea; 6 Department of Nursing, University of Ulsan, Ulsan, Republic of Korea; 7 Department of Biomedical Laboratory Science, Gimcheon University, Gimcheon, Republic of Korea

**Keywords:** autonomic nervous system, dyslipidemia, fibrosis, protocatechuic acid, syringin

## Abstract

**Background:**

Dyslipidemia causes complex cardiac pathologies associated with inflammation, fibrosis, and autonomic dysregulation. The prevention of heart damage induced by dyslipidemia and the mechanisms of action of the phenolic compounds protocatechuic acid and syringin remain incompletely elucidated.

**Purpose:**

This study investigated the role and mechanism of co-administration of two natural phenols, protocatechuic acid and syringin, previously isolated from *Saussurea neoserrata* Nakai, in improving cardiac damage in a high-fat diet-induced dyslipidemia mouse model.

**Method:**

To induce dyslipidemia in C57BL/6 mice, they were fed a high-fat diet for 4 weeks. Subsequently, while maintaining the high-fat diet, mice received daily administration of protocatechuic acid (50 mg/kg), syringin (50 mg/kg), a combination of both, or simvastatin (10 mg/kg) for 12 weeks. Subsequent evaluations include noninvasive electrocardiogram and heart rate variability assessment, Spearman correlation analysis, blood lipid and cytokine analysis, histopathological evaluation, immunohistochemistry, and Western blot.

**Result:**

Combination therapy with protocatechuic acid and syringin restored plasma lipid profiles, reduced plasma cytokines, and inhibited collagen accumulation and myocardial fibrosis in cardiac tissue. It also restored cardiac electrophysiological function and improved autonomic imbalance by alleviating sympathetic nervous system dominance. Mechanistically, it inhibited the TLR4/Myd88 inflammatory pathway and upregulated the Nrf2/HO-1 antioxidant pathway.

**Conclusion:**

This approach, which simultaneously targets fibrosis, inflammation, oxidative stress, and autonomic nervous system imbalance, demonstrates the potential for a multi-target therapeutic strategy against complex cardiac diseases caused by dyslipidemia.

## 1 Introduction

Dyslipidemia is a metabolic disorder characterized by abnormal levels of lipid components in the blood (Pappan et al., 2024). Discussed as a major cause of cardiovascular disease (CVD) progression, dyslipidemia (Graham et al., 2012) is clinically identified by decreased high-density lipoprotein (HDL) cholesterol and increased concentrations of triglycerides (TG), low-density lipoprotein (LDL) cholesterol, and total cholesterol (TC) (Jeppesen et al., 1997). These specific alterations in lipid metabolism are considered significant risk factors for CVD. Metabolic abnormalities such as dyslipidemia can cause changes in cardiac electrophysiological characteristics, potentially leading to electrocardiographic abnormalities such as QRS interval prolongation and QT interval changes (Li et al., 2023). Furthermore, endothelial dysfunction, induced by metabolic abnormalities, is associated with increased sympathetic nervous system activity, thought to arise from alterations in neurotransmitter secretion, reuptake, or receptor function (Lambert et al., 2013). A negative correlation was observed between LDL levels and autonomic nervous system HRV parameters in patients diagnosed with dyslipidemia (Pehlivanidis et al., 2001). This suggests that dyslipidemia may cause autonomic dysfunction. Previous studies indicate that dyslipidemia increases the risk of myocardial damage and fibrosis, and further exacerbates CVD (Ference et al., 2017; Elbadawi et al., 2024). Activation of collagen, one of the extracellular matrix proteins, and excessive accumulation of collagen within muscle fibers are characteristics of fibrosis (Frangogiannis, 2021). Moreover, the progression of fibrosis is closely associated with the inflammatory response (Thomas and Grisanti, 2020). Toll-like receptor 4 (TLR4) activates various downstream signaling pathways, including the nuclear factor kappa B (NF-κB) pathway. This receptor, which belongs to the pattern recognition receptor family, is a primary driver of the body’s inflammatory responses (Zhang and Ghosh, 2001). TLR4 stimulation activates NF-κB-mediated production of pro-inflammatory cytokines such as interleukin-1β (IL-1β), interleukin-6 (IL-6) and interleukin-18 (IL-18), while also amplifying the response to transforming growth factor-β (TGF-β) (Ben Ari et al., 2012; Bhattacharyya et al., 2013). A high-fat diet that causes dyslipidemia upregulates the concentration of lipopolysaccharide (LPS) in serum, and LPS promotes TLR4 activation (Guijarro-Muñoz et al., 2014; Zhu et al., 2015).

Protocatechuic acid (PCA, 3,4-dihydroxybenzoic acid) and syringin (SY) are natural phenolic compounds commonly found in plants. Previous reports have shown that PCA compounds have antioxidant, anti-inflammatory, and potential cancer-preventive activity (Lin et al., 2009; Tanaka et al., 2011). In addition, PCA improved cardiac autonomic imbalance and cardiac dysfunction in rats with type 1 diabetes (Semaming et al., 2014). Similarly, SY has been proven to have antinociceptive effects, and liver protecting effects (Choi et al., 2004; Gong et al., 2014). SY exerts anti-inflammatory and antioxidant effects and improves cardiac function, particularly by regulating the SIRT1 signaling pathway (Zhao et al., 2023). We previously isolated PCA and SY from *Saussurea neoserrata* Nakai for the first time. We demonstrated that PCA and SY protect cells through antioxidant and anti-inflammatory responses in human skin keratinocytes exposed to particulate matter (Jeong et al., 2023).

This study aims to evaluate the therapeutic potential of PCA and SY in mitigating cardiac damage caused by hyperlipidemia induced by a high-fat diet (HFD), based on their established anti-inflammatory and antioxidant properties.

## 2 Materials and methods

### 2.1 Animals

This study was conducted in accordance with the ARRIVE 2.0 guidelines. The experiment utilized eight-week-old male C57BL/6 mice (27 ± 2 g) sourced from Samtako (Osan, Republic of Korea). The animals were housed in a specific pathogen-free (SPF) environment. The environment maintained a temperature of 21 °C–24 °C, humidity of 45%–55%, and a 12 h light-dark cycle. The animals had unrestricted access to food and water throughout the experiment. The experimental animals were randomly divided into six groups, with six animals assigned to each group: Normal diet (Normal), High-fat diet (HFD), High-fat diet +50 mg/kg PCA (PCA), High-fat diet +50 mg/kg SY (SY), High-fat diet +50 mg/kg PCA and 50 mg/kg SY (PCA + SY), and High-fat diet +10 mg/kg simvastatin (SIM). The normal group was fed a diet containing 10% fat, while the high-fat diet consisted of 60% fat, 20% carbohydrates and 20% protein. The experiment was conducted over 16 weeks. After a 1-week adaptation period, the mice underwent a dietary induction period of 4 weeks on a normal diet or a high-fat diet. Immediately following the 4 weeks high-fat diet induction, the treatment group maintained a high-fat diet for 12 weeks while receiving oral administration of PCA, SY, their combination (PCA + SY), or SIM at the same time each day. Protocatechuic acid (PHL89766, Purity ≥98%, Sigma-Aldrich, St. Louis, MO, USA) and syringin (HY-N0824, Purity ≥98%, MedChemExpress, Monmouth Junction, NJ, USA) were dissolved in distilled water and administered, while the HFD group received distilled water alone. The identity and purity of the compounds were confirmed by the supplier through LCMS, HPLC, and NMR analysis. After the experiment concluded, animals were anesthetized using a mixture of 40 mg/kg Alfaxalone and 10 mg/kg Xylazine. Blood was then collected from the abdominal aorta, and the animals were sacrificed by cervical dislocation. The Investigators were not blinded to allocation during experiments and outcome assessment. Every effort was made to minimize animal suffering. No adverse reactions or deaths were observed during the study. Catholic University of Pusan Animal Experiment Ethics Committee Approval Number: CUP AEC 2023-006.

### 2.2 ECG and HRV analysis

After all mice had completed treatment, electrocardiograms (ECG) were measured. ECG was measured non-invasively while awake using ECGenie (Mouse Specifics, Inc., Framingham, MA, USA) equipment to minimize the effects of anesthetics. Prior to the ECG measurement, each mouse was given a 10 min period to acclimate to the recording apparatus. The same ECG signal as Eindhoven Lead I is generated when the mouse’s three paws touch the electrodes. After waiting for the mouse to enter a natural inactive state and 25-30 signals were recorded consecutively and then used for analysis. After confirming that peaks P, Q, R, and S were clear, they were used for analysis. The recorded signals were measured using the e-MOUSE program (Mouse Specifics, Inc., Framingham, MA, USA). The measured parameters were heart rate (HR), PR interval, QRS interval, QT interval and QTc interval, heart rate variability (HRV), low frequency (LF), high frequency (HF), LF/HF ratio, and root mean square of successive differences (rMSSD). The QTc interval was calculated by correcting the QT interval using the Bazett formula (Ahnve, 1985). LF power represents cardiac sympathetic and parasympathetic nerve activity, with a frequency range set at 0.15–1.5 Hz, while HF power represents cardiac parasympathetic nerve activity, with a frequency range set at 1.51–5 Hz (Just et al., 2000). Decreased HRV, increased LF/HF ratio, and decreased rMSSD associated with parasympathetic nervous system activity were used as measures of cardiac autonomic imbalance (Wang et al., 2024). ECG and HRV analyses were performed on five mice per group.

### 2.3 Histological staining

After harvesting the mouse hearts, they were immediately immersed in 10% buffered formalin for fixation. 4 μm thick sections were prepared from the paraffin-embedded fixed tissues. Prior to staining, these sections underwent deparaffinization with xylene and were rehydrated through a graded series of alcohol. Tissue slides were stained using standard procedures: hematoxylin-eosin (H&E), Masson’s trichrome for fibrosis assessment, and periodic acid-Schiff (PAS) for glycogen accumulation evaluation. Stained tissue images were visualized using a DMi1 (Leica, Wetzlar, Germany) microscope for randomly selected sections. We used ImageJ software used to quantify cross-sectional area (CSA) of cardiomyocytes and areas positive for Masson’s trichrome and PAS staining. Three non-contiguous sections were used per animal. Three random fields per sample were analyzed, and investigators were aware of group assignments.

### 2.4 Immunohistochemistry (IHC)

We performed IHC staining using the VECTASTAIN Elite ABC HRP kit and followed the manufacturer’s recommended protocol. Paraffin was removed using xylene and a series of alcohols, and then the antigen was revealed using antigen unmasking solution (Vector Laboratories, Newark, CA, USA). Hydrogen peroxide was used to block endogenous antigens, and the Anti-Collagen III antibody (1:200, abcam, Cambridge, UK) primary antibody was incubated overnight at 4 °C. All sections were visualized using a DMi1 microscope for randomly selected areas, and the stained areas were quantified using ImageJ software.

### 2.5 Western blot analysis

After collecting cardiac tissue, it was homogenized in RIPA solution containing protease and phosphatase inhibitors, centrifuged, and only the supernatant was obtained for use. 20 μg of protein per lane was loaded. The primary antibodies used in Western blot analysis are listed in Supplementary Table S1. Following protein transfer, the membrane was treated with a primary antibody solution overnight at 4 °C. The membrane was then incubated with anti-rabbit IgG secondary antibody (Cell Signaling Technology, Danvers, MA, USA) and protein detection was performed using an ECL kit (Amersham, Marlborough, MA, USA). The detected proteins were quantified using ImageJ software, and the final values were expressed as a ratio of the target protein to GAPDH.

### 2.6 Blood lipid parameters

Blood lipid parameters were measured after 24 h fast. Samples of blood were collected via the abdominal aorta from each mouse and maintained in heparinized tubes. Using a MNCHIP (Pointcare M4, Tianjin, China) biochemical analyzers, the blood markers HDL, LDL, TC and TG were quantified as specified in the manufacturer’s instructions.

### 2.7 Plasma cytokine analysis

Cytokine concentrations in blood were measured to evaluate markers of inflammation and fibrosis. The concentrations of cytokines (TGF-β, IL-1β, IL-6, IL-18) in the blood were measured using an ELISA kit from Abcam (Cambridge, UK) according to the recommended procedure.

### 2.8 Statistical analysis

We utilized GraphPad Prism 10 software to conduct one-way ANOVA with Tukey’s *post hoc* analysis for intergroup comparisons. Results are presented as mean ± standard deviation (SD), based on no fewer than three separate experimental runs, with *p* values under 0.05 considered statistically meaningful. All experiments were performed independently at least three times.

## 3 Results

### 3.1 PCA and SY improve cardiac dysfunction caused by dyslipidemia

**FIGURE 1:**
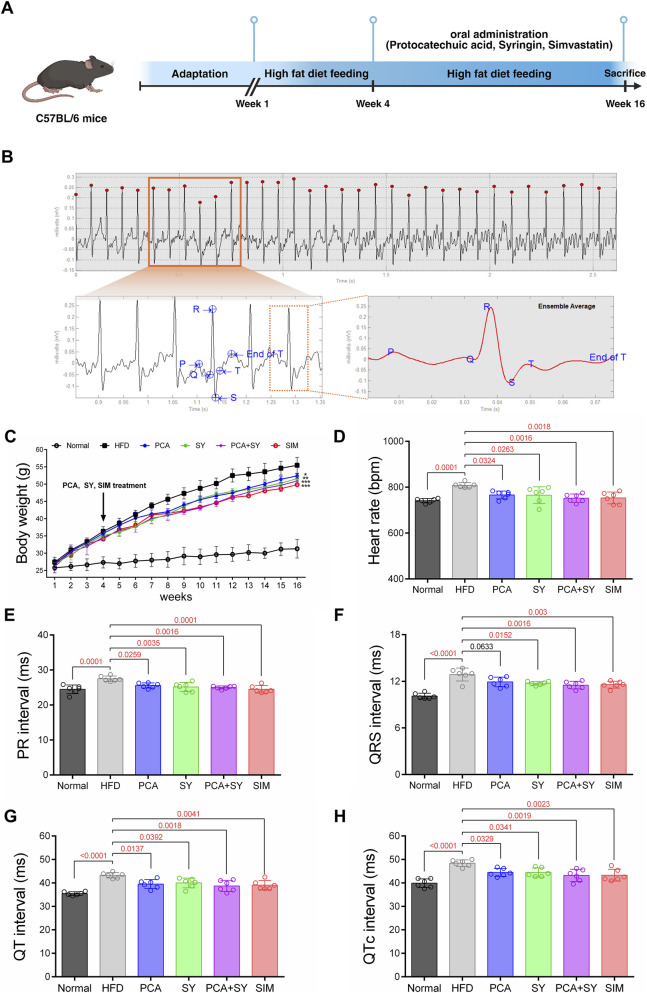
Evaluation of ECG parameters after PCA and SY treatment. **(A)** Schematic diagram of the study design for inducing dyslipidemia and treatment. Created with Biorender.com. **(B)** Original ECG recording of a representative mouse. **(C)** Measure weight changes every week. ECG parameters evaluated after various treatments **(D)** heart rate **(E)** PR interval **(F)** QRS interval **(G)** QT interval **(H)** QTc interval (*n* = 6). Data are presented as mean ± SD. For **(C–H)**, one-way ANOVA followed by Tukey’s *post hoc* test.

We initially provided a high-fat diet for 4 weeks, after which the animals received the same diet combined with treatment for another 12 weeks (Figure 1A). All treatment groups showed a significant effect on weight decrease compared to the HFD group (Figure 1C). We measured cardiac electrophysiological function and analyzed signals using noninvasive ECG (Figure 1B). The ECG measurement was performed while the mouse was conscious. ECG analysis results showed that the HFD group had significant cardiac electrical abnormalities compared to the normal group. Heart rate measurements showed relative bradycardia in the HFD group (HFD group: 741.1 ± 8.7 bpm, *p* = 0.0001) compared to the normal group (808.1 ± 8.7 bpm), and significant changes were also observed in ECG parameters. The PR interval was prolonged (27.5 ± 0.7 m, p = 0.0001), QRS interval was prolonged (13 ± 0.4 m, *p* < 0.0001), QT interval was prolonged (43.2 ± 1 m, p < 0.0001), and QTc interval was prolonged (48.4 ± 1.2 m, *p* < 0.0001) in the HFD group (Figures 1D–H). This shows that dyslipidemia (HFD) impairs cardiac electrophysiological function. However, when PCA, SY, or PCA + SY were administered, reductions in heart rate and prolongations of the PR interval (respectively 25.6 ± 0.7 m, *p* = 0.0259, 25.2 ± 1.2 m, *p* = 0.0035, 25.0 ± 0.4 m, *p* = 0.0016) QRS interval (respectively 12.1 ± 0.6 m, *p* = 0.0633, 11.8 ± 0.2 m, *p* = 0.0152, 11.5 ± 0.4 m, *p* = 0.0016), QT interval (respectively 39.6 ± 1.8 m, *p* = 0.0137, 40.0 ± 1.9 m, *p* = 0.0392, 38.7 ± 2.1 m, *p* = 0.0018), and QTc interval (respectively 44.5 ± 1.5 m, *p* = 0.0329, 44.5 ± 1.8 m, *p* = 0.0341, 43.2 ± 2.4 m, *p* = 0.0019) were significantly suppressed. Therefore, this demonstrates that PCA and SY treatment can prevent cardiac electrophysiological dysfunction.

### 3.2 PCA and SY improve parasympathetic function and heart rate variability in dyslipidemia mice

The HFD group showed a significant decrease in HRV and a significant decrease in HF compared to the normal group. In addition, the LF/HF ratio increased due to the decrease in HF, and the rMSSD value also decreased significantly. This indicates parasympathetic inhibition, a shift to sympathetic dominance, and autonomic imbalance. However, during PCA, SY and PCA + SY treatment, HRV and HF values increased significantly compared to the HFD group, the LF/HF ratio experienced a significant decrease due to the increase in HF, and rMSSD also increased significantly (Table 1). This suggests that administration of PCA and SY can alleviate HRV and autonomic imbalance caused by dyslipidemia.

**TABLE 1:** Heart rate variability evaluation.

Parameters	Normal	HFD	PCA	SY	PCA + SY	SIM
HRV	21.8 ± 3.77	9.08 ± 2.13^###^	15.16 ± 2.53*	15.06 ± 2.20*	16.54 ± 2.42**	17.36 ± 2.80**
LF (ms^2^)	0.93 ± 0.41	0.95 ± 0.23	0.73 ± 0.30	0.74 ± 0.27	1.01 ± 0.27	0.95 ± 0.25
HF (ms^2^)	1.51 ± 0.38	0.43 ± 0.09^###^	0.51 ± 0.19	0.53 ± 0.21	1.05 ± 0.35*	1.02 ± 0.27*
LF/HF ratio	0.61 ± 0.24	2.20 ± 0.31^###^	1.49 ± 0.52*	1.45 ± 0.23*	0.98 ± 0.11***	0.95 ± 0.16***
rMSSD (ms)	3.02 ± 0.77	1.36 ± 0.19^##^	2.00 ± 0.40	2.09 ± 0.30	2.68 ± 0.55*	2.66 ± 0.73*

n = 5, data are presented as mean ± standard deviation (SD).

^##^ *p* < 0.01.

^###^ *p* < 0.001 versus normal group.

^*^ *p* < 0.05.

^**^ *p* < 0.01.

^***^ *p* < 0.001 versus HFD, group.

### 3.3 PCA and SY improve plasma lipid parameters caused by dyslipidemia

**FIGURE 2:**
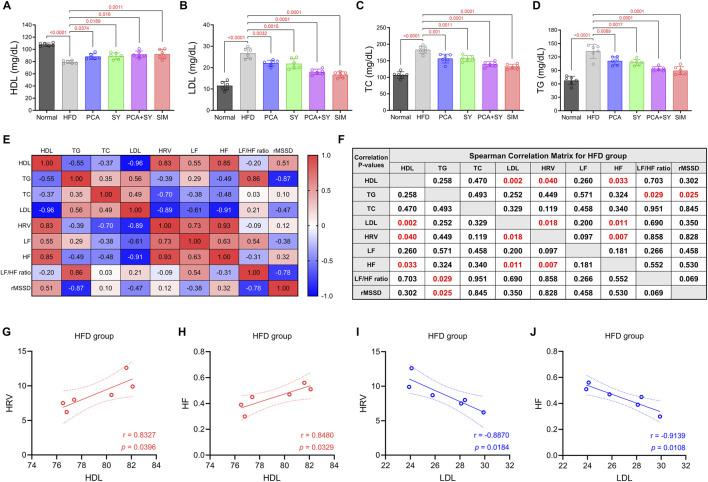
Effect of PCA and SY treatment on plasma lipid parameters and Spearman correlation analysis. Quantification of plasma **(A)** HDL **(B)** LDL **(C)** TC **(D)** TG expression levels (*n* = 6). Correlation between plasma lipid parameters and HRV parameters in HFD group mice **(E)** r value and **(F)** *p* value (*n* = 6). **(G–J)** Correlation analysis between HRV parameters (HRV, HF) and lipid parameters (HDL, LDL) (*n* = 6). Solid lines and dotted lines represent linear regression lines and 95% confidence intervals. Data are presented as mean ± SD. For A-D, one-way ANOVA followed by Tukey’s *post hoc* test; for E-J, two-tailed t-test for Spearman’s correlation analysis.

A high-fat diet was confirmed through various evaluations to disrupt the lipid profile and cardiac autonomic regulation in mice. Compared to the normal group, HDL levels were significantly reduced in the HFD group (79.1 ± 2.3 mg/dL, *p* < 0.0001) (Figure 2A), while LDL (26.7 ± 2.3 mg/dL, *p* < 0.0032), TC (183.3 ± 10.4 mg/dL, *p* < 0.0001), and TG (132.7 ± 14.1 mg/dL, *p* < 0.0001) levels were significantly increased (Figures 2B–D). This shows that a high-fat diet caused dyslipidemia. However, administration of PCA, SY and PCA + SY increased HDL (respectively 88.2 ± 4.1 mg/dL, *p* = 0.0374, 89.0 ± 4.7 mg/dL, *p* = 0.0189, 91.7 ± 5.2 mg/dL, *p* = 0.0011) and decreased LDL (respectively 22.1 ± 1.3 mg/dL, *p* = 0.0032, 21.3 ± 2.5 mg/dL, *p* = 0.0015, 18.1 ± 1.2 mg/dL, *p* = 0.0001), TC (respectively 156.7 ± 12.3 mg/dL, *p* = 0.001, 154.7 ± 10.5 mg/dL, *p* = 0.0011, 139.7 ± 6.2 mg/dL, *p* = 0.0001), and TG (respectively 111.2 ± 8.3 mg/dL, *p* = 0.0089, 105.3 ± 7.0 mg/dL, *p* = 0.0017, 93.8 ± 4.5 mg/dL, *p* = 0.0001), resulting in significant improvement in all lipid parameters. We also investigated the correlation between lipid parameters and the autonomic nervous system. In Spearman correlation analysis (Figures 2E,F), HDL in the HFD group showed a significant positive correlation with HRV (r = 0.8327, *p* = 0.0396) and HF (r = 0.8480, *p* = 0.0329) (Figures 2G,H). Conversely, LDL showed a significant negative correlation with HRV (r = −0.8870, *p* = 0.0184) and HF (r = −0.9139, *p* = 0.0108) (Figures 2I,J). Improvements in lipid profiles showed a significant correlation with the alleviation of HRV parameters. These results suggest a potential association between lipid reduction and autonomic nervous system recovery.

### 3.4 PCA and SY improve myocardial tissue pathology caused by dyslipidemia and inhibit collagen accumulation

**FIGURE 3:**
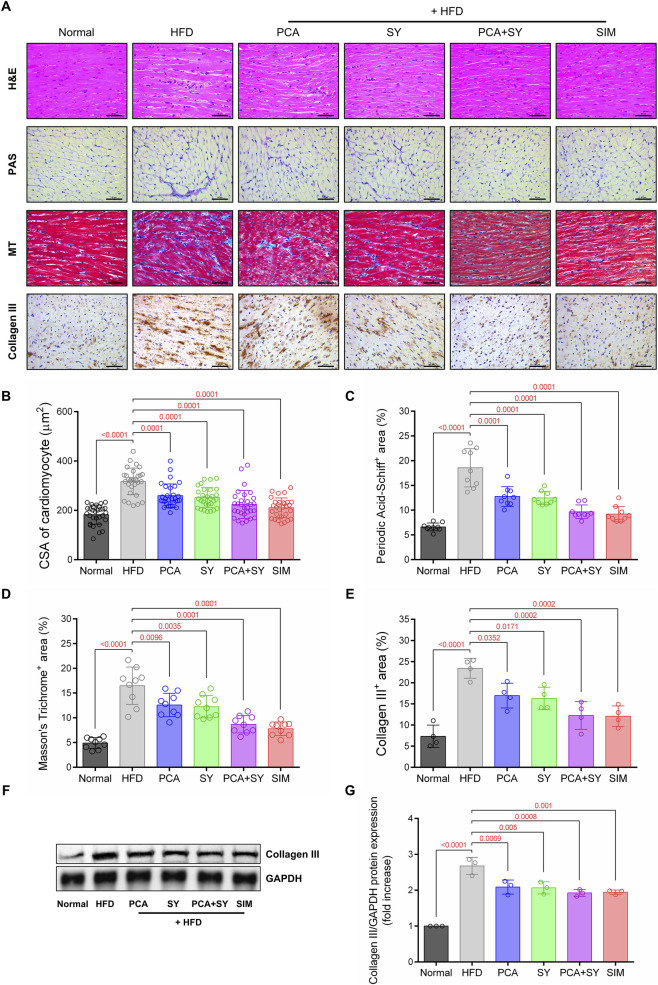
Effects of PCA and SY treatment on cardiac fibrosis. **(A)** Representative histological staining of H&E, PAS, MT, and collagen III immunohistochemical staining. Scale bar = 50 μm. **(B)** Quantification of cross-sectional length of cardiac muscle cells (*n* = 30). **(C)** Quantitative analysis of areas positive for periodic acid-Schiff staining in cardiac tissue (*n* = 9). **(D)** Quantification of the positive area of Masson’s trichrome staining in cardiac tissue (*n* = 9). **(E)** Quantitative analysis of collagen III immunohistochemical staining positive areas in cardiac tissue (*n* = 4). **(F)** Western blotting for collagen III protein expression in cardiac tissue. **(G)** Quantification of collagen III protein expression (*n* = 3). Data are presented as mean ± SD. For B-E, G, one-way ANOVA followed by Tukey’s *post hoc* test.

Histopathological changes and collagen deposition were evaluated using H&E, PAS, MT staining, and Collagen III IHC of cardiac tissue (Figure 3A). Histological analysis revealed that dyslipidemia induces significant changes in cardiac structure. The HFD group exhibited increased cardiomyocyte CSA and wider interstitial spaces, as observed through H&E staining. However, PCA + SY treatment suppressed the increase in cross-sectional area (CSA) (Figure 3B). PAS staining analysis revealed a significant increase in periodic acid-Schiff-positive areas indicating glycogen accumulation in the HFD group (Figure 3C). Treatment with PCA, SY, or a combination of both significantly reduced this glycogen accumulation, with the combination therapy showing the most significant reduction. Additionally, the HFD group exhibited significantly higher myocardial fibrosis areas as confirmed by Masson’s trichrome staining, and it was confirmed that fibrosis areas were significantly downregulated by treatment with PCA, SY, or combination therapy (Figure 3D). Collagen III expression was confirmed via IHC and Western blot analysis (Figures 3E–G). The HFD group exhibited a substantial increase in collagen III expression, whereas treatment with PCA, SY, or combination therapy resulted in a substantial reduction in collagen III expression. This suggests that PCA + SY treatment alleviated pathological changes and fibrosis in cardiac tissue.

### 3.5 PCA and SY suppress inflammation in cardiac tissue caused by dyslipidemia

**FIGURE 4:**
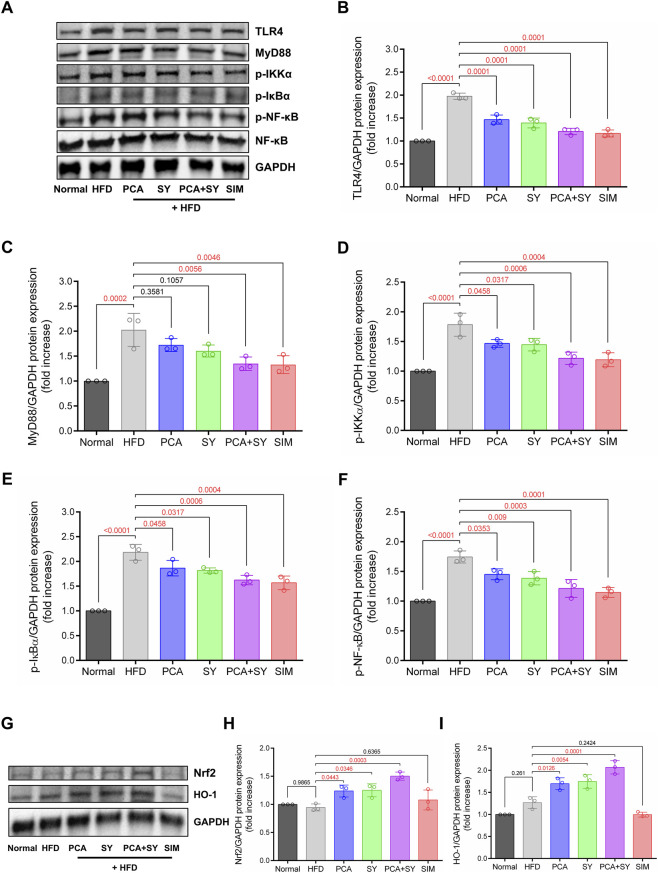
Effects of PCA and SY treatment on TLR4/NF-κB and Nrf2/HO-1 pathways. **(A)** Western blot analysis of TLR4, MyD88, phosphorylated IKKα, phosphorylated IκBα, phosphorylated NF-κB, and NF-κB protein expression in cardiac tissue. Quantification of **(B)** TLR4, **(C)** MyD88, **(D)** p-IKKα, **(E)** p-IκBα, and **(F)** p-NF-κB protein expression (*n* = 3). **(G)** Western blot analysis of Nrf2/HO-1 protein expression in cardiac tissue. Quantification of **(H)** Nrf2 and **(I)** HO-1 protein expression (*n* = 3). Data are presented as mean ± SD. For B-F, H, I, one-way ANOVA followed by Tukey’s *post hoc* test.

Western blot analysis confirmed the molecular mechanisms of PCA and SY in dyslipidemia-induced cardiac inflammatory response. In Figures 4A–F, we confirmed that the TLR4/MyD88 signaling pathway was activated in heart tissue samples from the HFD group, IKKα and IκBα phosphorylation increased, and NF-κB activation, a key factor in inflammatory responses, was promoted. PCA and SY administration led to a significant decrease in TLR4 and MyD88 protein levels in cardiac tissue. In addition, the inhibition of IKKα and IκBα phosphorylation suggests that inflammatory responses can be improved through decreased NF-κB activation. Additionally, the HFD group had decreased protein levels of Nrf2 and HO-1. However, PCA and SY treatment significantly increased these expressions (Figures 4G–I). PCA and SY attenuate NF-κB-mediated inflammatory responses, which is associated with the regulation of the TLR4/MyD88 signaling pathway induced by dyslipidemia. Furthermore, the increase in antioxidant activity associated with the Nrf2-HO-1 signaling pathway suggests potential cardioprotective effects of these treatments.

### 3.6 PCA and SY improve plasma inflammatory cytokine levels

**FIGURE 5:**
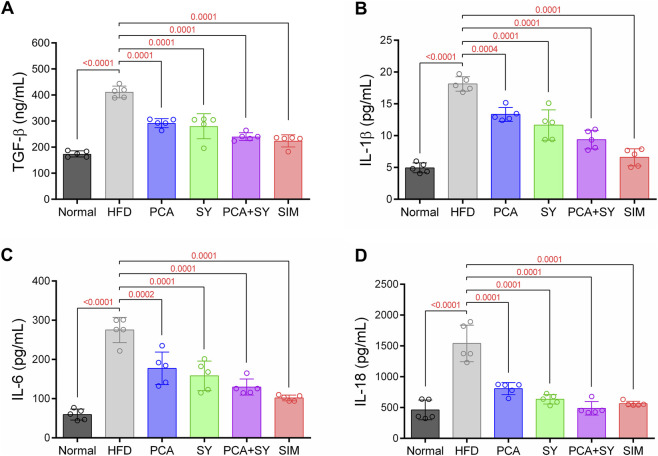
Effects of PCA and SY treatment on inflammatory cytokines in plasma. Quantification of plasma **(A)** TGF-β **(B)** IL-1β **(C)** IL-6 **(D)** IL-18 expression levels (*n* = 5). Data are presented as mean ± SD. For A-D, one-way ANOVA followed by Tukey’s *post hoc* test.

Compared to the normal group, plasma levels of inflammatory cytokines TGF-β, IL-1β, IL-6, and IL-18 were significantly increased in the HFD group (Figures 5A–D). However, treatment with PCA and SY significantly decreased the expression of inflammatory cytokines. This suggests that PCA and SY treatment suppress plasma inflammatory cytokines induced by hyperlipidemia caused by a high-fat diet.

## 4 Discussion

In this study, the cardioprotective effects of PCA and SY on heart dysfunction induced by dyslipidemia caused by a high-fat diet in C57BL/6 mice were investigated. The results showed that oral administration of PCA and SY improved ECG parameters, decreased cardiac fibrosis, downregulation of the TLR4/MyD88 signaling pathway and upregulation of the Nrf2/HO-1 signaling pathway via Western blot analysis, improvement of plasma lipid parameters, and decreased plasma inflammatory cytokines.

The observed improvements in lipid profiles and ECG parameters underscore the role of phenolic compounds in regulating metabolic disorders. PCA and SY are known as phenolic compounds (Kakkar and Bais, 2014; US et al., 2015). We established a dyslipidemia model by feeding mice a high-fat diet, and administration of PCA and SY improved the plasma lipid profile. Phenolic acid PCA has been reported to improve lipid metabolism by inhibiting cholesterol absorption in mouse models. Similarly, the decrease in SY’s lipid parameters aligns with previous research findings showing increased HDL and decreased LDL, TC, and TG in diabetic mouse models, and may contribute to these effects (Deng et al., 2023). Our findings align with the known biological activities of phenolic compounds and indicate that the cardioprotective effects are linked to their capacity to improve lipid parameters.

PCA and SY influence cardiac electrophysiological function and cardiac autonomic regulation. The HFD group exhibited significant ECG abnormalities compared to the normal group, including bradycardia and prolongation of the PR, QRS, QT and QTc intervals. These changes are associated with cardiac conduction and ventricular repolarization disorders, which are known risk factors for severe heart disease (Watanabe et al., 2008). However, the combination of PCA and SY prevented these changes and demonstrated a cardioprotective or regulatory effect on the cardiac electrical system. This is further supported by HRV analysis of autonomic nervous system status. In the HFD group, the LF/HF ratio increased, indicating sympathetic dominance, and HF and rMSSD, which are key indicators reflecting parasympathetic activity, decreased. Such autonomic nervous system imbalance is well known to be a cause of CVD incidence (Malpas, 2010; Thayer et al., 2010). Combination therapy with PCA and SY restored autonomic homeostasis by increasing HF levels and significantly decreasing the LF/HF ratio. Based on previous reports, this demonstrates a powerful cardioprotective mechanism in which vagal tone recovery is associated with improved cardiac elasticity (Liu et al., 2015; Mesquita et al., 2017; Liu et al., 2019; Hosseini et al., 2025). However, there are inherent limitations to interpreting HRV parameters in mice. Unlike humans, the LF component in mice reflects regulation from both the sympathetic and parasympathetic nervous systems, making the LF/HF ratio an indirect indicator for estimating autonomic nervous system balance. Furthermore, ECG recordings during conscious states are highly sensitive to subtle changes in physical activity and respiration.

In addition to physiological improvements, the TLR4/MyD88 and Nrf2/HO-1 signaling pathways are associated with inflammatory CVD and play important roles (Yang et al., 2016; Raish et al., 2022). The results of this study suggest that activation of the TLR4/MyD88 signaling pathway can regulate the inflammatory response in dyslipidemic mice. Consistent with this, we confirmed that the TLR4/MyD88 signaling pathway activates NF-κB phosphorylation, which is associated with increased levels of the inflammatory cytokines IL-1β, IL-6, and IL-18 (Youssef et al., 2021). This persistent inflammation causes myocardial fibrosis (Suthahar et al., 2017). Previous reports have shown that hypertriglyceridemia, which destroys the antioxidant enzyme defense system, can cause cell and tissue damage (Hong et al., 2009). Such circulating lipid excess leads to excessive production of reactive oxygen species (ROS), overwhelming the heart’s endogenous antioxidant capacity and causing cardiovascular pathology (Chen and Maltagliati, 2018). When intracellular ROS levels increase, the Keap1 protein dissociates from Nrf2 (Tu et al., 2019). Consequently, Nrf2 is able to translocate to the nucleus, where it acts as a key regulator of gene transcription, stimulating the synthesis of protective proteins such as antioxidant enzymes and HO-1 (Pan et al., 2016). The activation of the Nrf2 pathway has been proven in many studies to have a cardioprotective effect (Jiang et al., 2016). In this study, we demonstrated that PCA and SY can alleviate the inflammatory response to dyslipidemia by downregulating the TLR4/MyD88 pathway. It also suggests that it decreases cardiac damage by upregulating Nrf2 and HO-1 expression.

ELISA analysis results showed that TGF-β, IL-1β, IL-6, and IL-18 levels increased in dyslipidemia induced by a high-fat diet. Increased levels of these inflammatory cytokines have been associated with cardiac hypertrophy, fibrosis, and myocardial damage. (Fix et al., 2011; Peiró et al., 2017; Kumar et al., 2019). However, treatment with PCA and SY showed downregulation of inflammatory cytokines. The anti-inflammatory properties observed in PCA and SY are consistent with findings from previous studies (Lin et al., 2009; Shen et al., 2020). Additionally, the results confirmed in this study demonstrate an inhibitory effect on cardiac fibrosis. The antifibrotic effects of PCA and SY suppressed fibrosis formation by decreasing glycogen and collagen accumulation. This antifibrotic effect may involve downregulation of TGF-β levels, which is similar to previous findings showing that PCA alleviated fibrosis in a hepatic fibrosis model by reducing TGF-β levels (Cui et al., 2021).

Despite the cardioprotective effects of PCA and SY observed in this study, several limitations exist. This study may not encompass the full range of biological variation inherent in complex disease models due to its small sample size. Additionally, we used only male C57BL/6 mice. Noninvasive ECG and HRV measurements provide useful electrophysiological information, but cardiac function imaging tests such as echocardiography were not performed. Therefore, the results of this study are primarily limited to electrophysiological characteristics. Additionally, applying this to actual clinical settings requires a cautious approach that accounts for interspecies differences and pharmacokinetic variations between mice and humans.

In this study, PCA 50 mg/kg and SY 50 mg/kg were administered orally. These doses were selected based on prior research demonstrating the efficacy of PCA and SY in mitigating oxidative stress and inflammatory damage in rodent models (Lende et al., 2011; Shen et al., 2020). In this study, although toxicological indicators or general health indicators were not monitored during the 12-week treatment period, no deaths or signs of toxicity were observed. From a translational perspective, calculating the human equivalent dose (HED) reveals that our mouse dose corresponds to approximately 4 mg/kg in humans, a concentration clinically achievable through supplements (Shin et al., 2010). However, the low oral bioavailability and rapid metabolic rate of phenolic compounds remain significant challenges in translational estimation. To improve the therapeutic index of these compounds in the clinical setting, further research on optimized delivery systems or nanoformulations may be necessary (Esfanjani et al., 2018).

This study demonstrated that the antioxidant effects and cardiac autonomic regulation caused by the inhibition of the TLR4/MyD88 pathway and the activation of the Nrf2/HO-1 pathway due to PCA and SY treatment alleviate cardiac damage caused by dyslipidemia. Simvastatin, a traditional dyslipidemia treatment, works by competitively inhibiting HMG-CoA reductase, which directly blocks cholesterol synthesis (Istvan and Deisenhofer, 2001). However, PCA and SY demonstrated cardioprotective effects by targeting complex pathological mechanisms such as anti-fibrosis, anti-inflammation, anti-oxidation, and autonomic nervous system regulation, rather than directly targeting the cholesterol synthesis pathway. This complementary combination of PCA and SY suggests that it could be a useful adjunct or alternative to lipid-lowering therapy alone in the management of CVD.

## 5 Conclusion

In conclusion, PCA and SY effectively alleviate dyslipidemia-induced cardiac injury and autonomic imbalance. These cardioprotective effects are mediated through inhibition of the TLR4/MyD88 inflammatory pathway and activation of the Nrf2/HO-1 antioxidant defense pathway. This multi-target approach, simultaneously targeting inflammation, oxidative stress, and fibrosis, presents a therapeutic strategy for managing the complex cardiac pathologies associated with dyslipidemia.

## Data Availability

The raw data supporting the conclusions of this article will be made available by the authors, without undue reservation.
