# Supplementary material for: Protocatechuic acid and syringin improve cardiac damage and autonomic imbalance caused by dyslipidemia in mice
Source: Front Pharmacol. 2026 May 18;17:1809711. doi: 10.3389/fphar.2026.1809711 (PMC13222979; doi:10.3389/fphar.2026.1809711)
Supplement: Supplementary file 1 [file Table1.docx]

Supplementary Material

# Supplementary Table

**Table S1. Primary antibodies used in western blot**

| **Protein** | **Dilution** | **Cat No.** | **Manufacturer** |
| --- | --- | --- | --- |
| GAPDH (35kDa) | 1:10,000 | 2118 | Cell signaling |
| TLR4 (100kDa) | 1:1,000 | A11226 | ABclonal |
| MyD88 (33kDa) | 1:1,000 | Ab219413 | abcam |
| p-IKKα (85kDa) | 1:1,000 | 2697 | Cell signaling |
| p-IκBα (35kDa) | 1:1,000 | 2859 | Cell signaling |
| p-NF-κB (65kDa) | 1:1,000 | AP1294 | ABclonal |
| NF-κB (65kDa) | 1:1,000 | A19653 | ABclonal |
| Anti-collagen III (150kDa) | 1:1,000 | ab184993 | abcam |
| Nrf2 (100kDa) | 1:1,000 | 12721 | Cell signaling |
| HO-1 (28kDa) | 1:1,000 | 43966 | Cell signaling |
